# Cystic lesions in giant intraventricular meningioma: Two case reports

**DOI:** 10.1097/MD.0000000000032494

**Published:** 2023-02-22

**Authors:** Hai Yu, Junhua He, Min Yang

**Affiliations:** a Department of Neurosurgery, The Affiliated Hospital of Hangzhou Normal University, Hangzhou, China; b Department of Neurosurgery, Tongde Hospital of Zhejiang Province, Hangzhou, China; c Department of Ophthalmology, Tongde Hospital of Zhejiang Province, Hangzhou, China.

**Keywords:** atypical, cystic lesions, giant, intraventricular meningioma

## Abstract

**Rationale:**

Giant meningioma stemming from the intraventricular zone has been reported to be extremely few. Two cases of supersize (>8 cm) intraventricular meningiomas presenting with cystic lesions and their clinical characteristics were collected in our study.

**Patient concerns:**

One patient was a 56-year-old man who was hospitalized for blunt headache and weakness of the right lower limb along with a defect of the right visual field for 12 months. The other patient is a 22-year-old lady who presented with a slight headache accompanied by right facial numbness for 1 week. None of them had a significant past medical history.

**Diagnoses:**

Computed tomography and magnetic resonance imaging of both patients revealed a giant heterogeneous, enhancing tumor mainly in the left trigonum with low-density or hypointense cystic lesions located within or around the tumor. The pathological and immunohistochemical staining indicated fibroblastic meningioma (case 1) and atypical meningioma (case 2) respectively, thus the patients’ diagnoses were confirmed.

**Interventions:**

Total microsurgical resection, including the cystic wall, was performed via a transcortical (transtemporoparietal occipital) approach in both patients, and a ventricular drainage tube was placed for 24 to 48 hours routinely after removed the tumor.

**Outcomes:**

Postoperatively, both patients recovered free from episodes of symptoms, and imaging examinations confirmed no evidence of regrowth of the meningioma during an average 24 months follow-up.

**Lessons:**

Cystic lesions may indicate the histopathologic malignancy of intraventricular meningioma. Transcortical approach through the posterior temporal lobe or the parieto-occipital lobe is an effective technology for giant intraventricular meningiomas.

## 1. Introduction

Meningiomas are the most common nonmalignant intracranial tumors with an incidence of 37.6%,^[1,2]^ while they involving the intraventricular space are rare, which account for only 0.5% to 5% of intracranial meningiomas.^[3]^ Intraventricular meningiomas (IVMs) are usually described as an insidious enlargement of the size leading to generalized hydrocephalus disorders or neurological mass effects, which are characterized highly by location and histopathologic features. The most common lesion site of IVMs is the trigone area of the lateral ventricle.^[4,5]^ Being initially “silent,” they could be inconceivably large in size before hospitalization, representing a particular challenge for surgical procedures.^[6,7]^

In this paper, we collected the pathomorphological characteristics, surgical treatment, and outcome of 2 cases of oversize IVM (>8 cm) with cystic degeneration, which is extremely rare and has not been reported yet as far as we know, so as to improve the understanding of this sub-type of IVMs. This study was approved by the Clinical Research and Ethics Committee at the Affiliated Hospital of Hangzhou Normal University, and written consent were obtained from the patients for publication.

## 2. Case report

### 2.1. Clinical presentation

**Figure 1.**
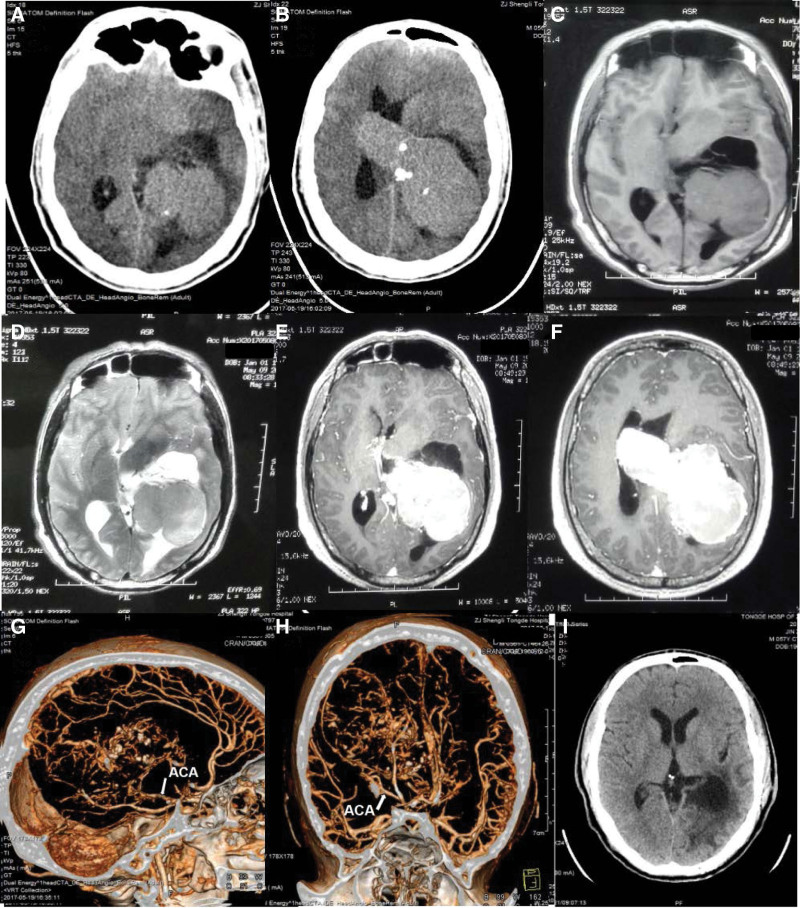
(A–I) Radiographic features of case 1 in the perioperative period. (A and B) CT scans revealed a super size heterogeneously irregular mass in the bilateral ventricle with peritumoral low-density cystic lesions. (C and D) Noncontrast-enhanced MRI scans showed solid part of the tumor was isointense on T_1_WIs, slightly hyperintense on T_2_WIs, and the cystic portion of the tumor was hypointense on T_1_WIs and hyperintense on T_2_WIs. (E and F) Contrast-enhanced T_1_WIs suggested significant enhancement of tumoral parenchyma, but non-enhanced of cyst wall. (G and H) CTA indicated an affluent blood supply of the tumoral parenchyma stemming from ACA. (I) Postoperative CT demonstrated total resection of the tumor. ACA = anterior choroidal artery, CT = computed tomography, CTA = computed tomographic angiography, MRI = magnetic resonance imaging, T_1_WIs = T_1_ weighted sequence, T_2_WIs = T_2_ weighted sequence.

**Figure 2.**
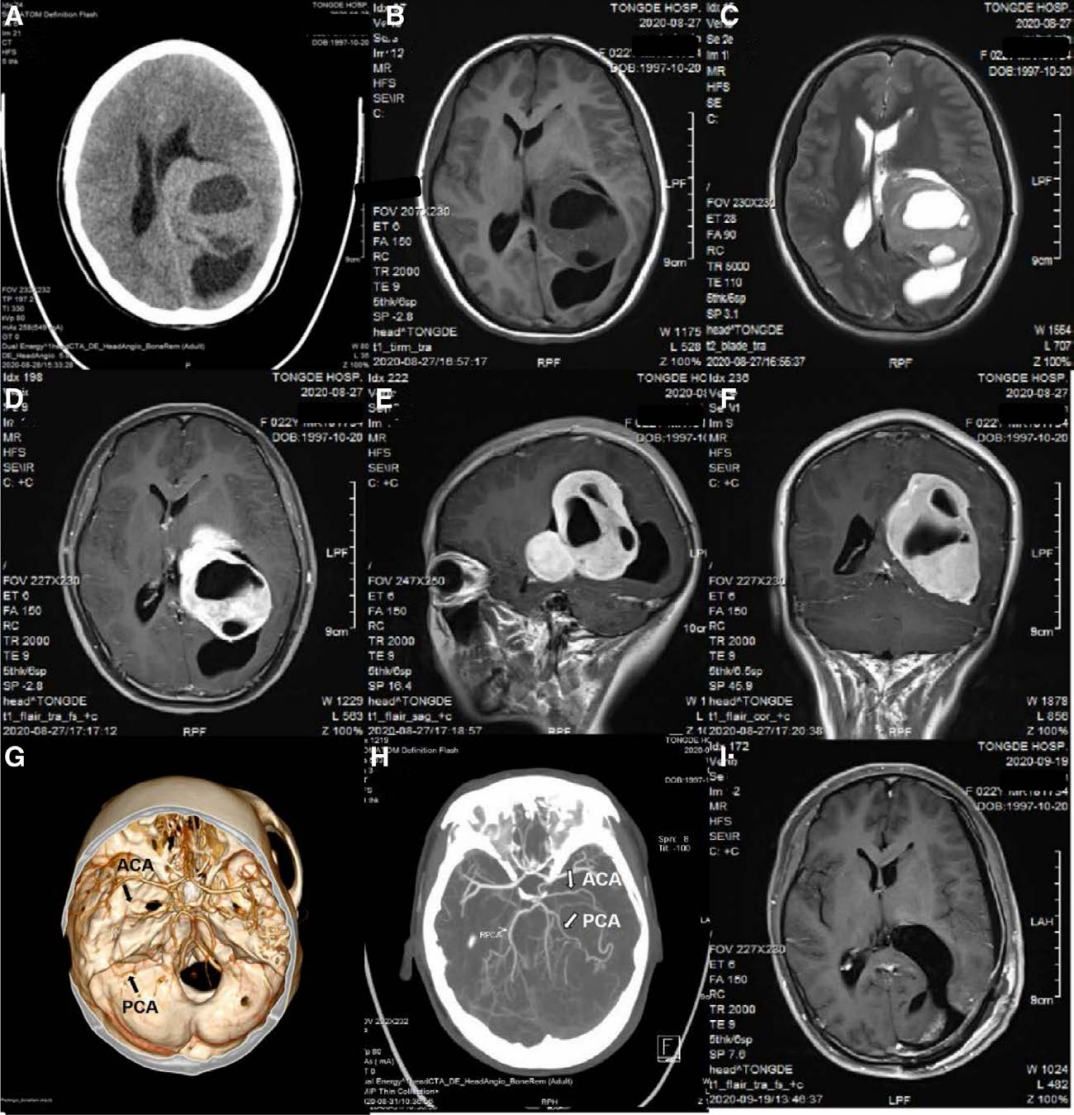
(A–I) Radiographic features of case 2 in the perioperative period. (A) CT scans revealed an extremely large mass in the left lateral ventricle with intratumoral multiple low-density cystic lesions. (B–F) MRI scans showed similar imaging features of the tumor on T_1_ weighted sequence, T_2_WIs, and the contrast-enhanced T_1_WIs following the injection of a contrast medium. (G and H) CTA indicated an affluent blood supply stemming from ACA and PCA. (I) Postoperative contrast-enhanced T_1_WIs demonstrated total resection of the tumor. ACA = anterior choroidal artery, CT = computed tomography, CTA = computed tomographic angiography, MRI = magnetic resonance imaging, PCA = posterior choroidal artery, T_1_WIs = T_1_ weighted sequence, T_2_WIs = T_2_ weighted sequence.

The first patient was a 56-year-old man, who complained of blunt headache and weakness in the right lower limb along with a defect of the right visual field for 12 months. The second patient was a 22-year-old female who presented with a slight headache accompanied by right facial numbness for 1 week. The system disorder history was negative in both cases. The computed tomography (CT) examination of both patients demonstrated radiographic features of a giant iso-density, peritumoral/intratumoral low-density cystic lesions mass (Fig. 1A and B and Fig. 2A) mainly in the left lateral ventricle (Nauta^[8]^ type II and type I), and CT angiography indicated an affluent blood supply stemming from choroidal artery (Fig. 1G and H and Fig. 2G and H). The magnetic resonance imaging (MRI) showed that the solid part of the tumor was isointense on T1-weighted images, slightly hyperintense on T2-weighted images compared with brain tissue (Fig. 1C and D and Fig. 2B and C), and enhanced MRI revealed well-demarcated dimension of 10.5 × 6.2 × 6.1 cm and 8.5 × 6.4 × 6.2 cm respectively in left intraventricular mass with midline shift in both patients (Fig. 1E and F and Fig. 2D–F).

### 2.2. Surgical management and outcome

**Figure 3.**
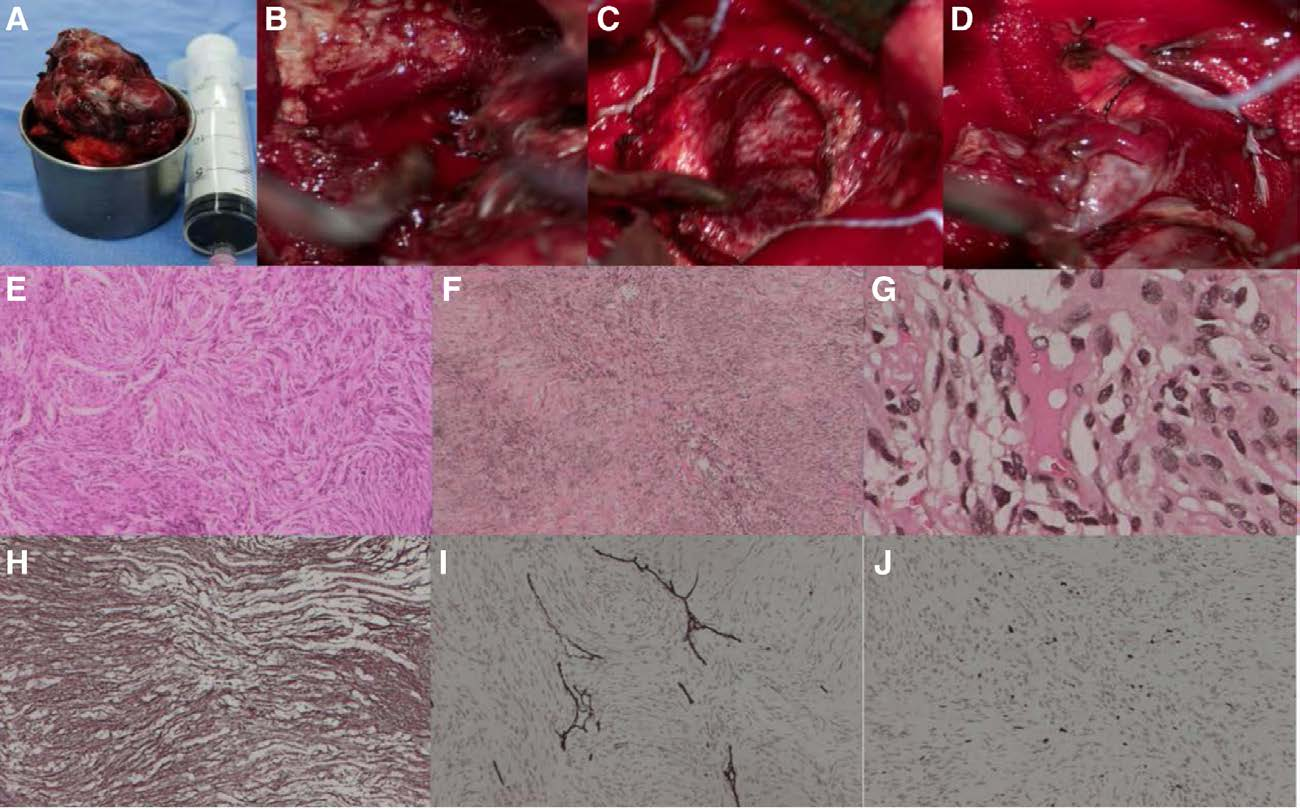
(A–J) Intraoperative exhibition and postoperative features of histopathology. (A) Gross specimen of tumor, (B–D) necrosis, cystic cavity and wall, and feeding artery (ACA) of the tumor under the microscope. (E) The fibroblastic pattern of the tumor in patient 1 (H&E, ×50, WHO I). (F and G) local infarction, necrotic foci, and mitotic figures of the tumor were obviously observed in patient 2 (H&E, ×50, ×400, WHO II). (H–J) the tumor strongly expresses VIM, CD34, Ki-67, etc by immunohistochemical staining (×200). ACA = anterior choroidal artery, H&E = hematoxylin and eosin, VIM = vimentin, WHO = World Health Organization.

An entire resection procedure through temporo-occipital approach was performed in case 1 and a parieto-occipital approach in case 2, the mean operative time was 7.5 hours. The cortical incision was determined by the distance between the cortex and the tumors, and the direction of long axis and growth of the tumors, paying equal attention to the protection of the functional structures. When the tumor was exposed, the operative field was expanded with the assistance of a brain retractor. The tumor was primarily excised in blocks within the capsule under the microscope, followed by the peritumoral separation and tumor-feeding artery occlusion (Fig. 3B–D). We observed that the tumors were elastic and had a rich vascularity that favored growth and invasion, and seemed to arise from the choroid plexus of trigone. A ventricular drainage tube was placed for 24 to 48 hours routinely after removed the tumor. At a mean follow-up of 24 months, both patients were neurologically intact and reported no further episodes of symptoms and CT/MRI showed no evidence of regrowth of the meningioma (Figs. 1I and 2I).

### 2.3. Histopathology

The gross appearance of both tumors was grayish-red soft tissue fragments (Fig. 3A). Histological examination demonstrated a fibroblastic meningioma in patient 1 while an atypical meningioma in patient 2. Hematoxylin and eosin staining revealed a multi-vascularity tumor biopsy, interpenetrating thin-walled dilated vessels in both patients. Additionally, infarction of partial tumor tissue, necrotic foci, and mitotic figures was obviously observed in patient 2 (Fig. 3F–G). Immunohistochemical labeling showed active expression of Ki-67 (2% and 5% respectively), positive for vimentin and epithelial membrane antigen in both patients (Fig. 3H–J); Besides, S-100 and P53 were positive in patient 2. Accordingly, the tumor diagnosis was consistent with World Health Organization^[9]^ grade I in patient 1 and grade II in patient 2.

## 3. Discussion

Cystic meningioma is uncommon and accounts for 1.7 to 11.7% of all intracranial meningiomas, which is more inclined to occur in children than in adults^[10–12]^; besides, they were reported mostly in the cerebral convexity and parasagittal areas, but rare in the intraventricular region.^[11,12]^ Thus, the cystic IVMs may be highly misdiagnosed as ependymomas, choroid plexus papillomas, or neurocytomas, which are more prone to appear with lesions of cysts and calcification in the ventricular zones. In the absence of histopathological examination, we know of no way to differentiate the cystic lesions with certainty by radiographic means.

Notably, the current 2 cases demonstrated histologically different sub-types, which may be associated with their various pathomorphological features. The first case was a fibroblastic meningioma, filled with mesenchymal elements, including gelatinous fiber, osseous and myxoid tissue, which may lead to calcification or ossification. In contrast, the second case was of an atypical meningioma, which is characterized by more blood supply, larger cyst cavity, tumoral infarction, necrotic foci, and mitotic figures, and higher positive rate of tumor immune markers, such as Ki-67, S-100, P53, etc. Consequently, we considered that the pathomorphological mechanisms of cystic changes in current cases may be correlated to the secretion activity of the cystic tumor cells, infarction and necrosis of tumor tissue, peripheral brain edema, and loculated widened subarachnoid space.

Total resection of microsurgery for such giant IVMs which straddle the bilateral ventricles remains a considerable challenge because of deep location, bleeding control, and operative perseverance, especially in the dominant hemisphere that is in close proximity to important structures, and even most patients have minimal deficits before hospitalization. Therefore, based on the tumor morphology and location, giving consideration to minimizing the impairments of eloquent and visual structures and brain retraction around the surgical access, we employed transcortical approaches through the posterior temporal lobe in patient 1 and the parieto-occipital lobe in patent 2. Additionally, in our experience, these routes are often the shortest paths to the tumors and make it possible for earlier control of the feeding vessels, appearing to be the most popular to trigonal IVMs.^[5–7,13,14]^

In order to achieve the goal of complete resection and reduce the risk of recurrence, the neurosurgeon should make a reasonable procedure to cyst walls according to the individual situation of patients and could never peel off the wall blindly.^[12,14,15]^ On the basis of the cyst location and the relationship between the tumor and the adjacent brain, Nauta^[8]^ classified cystic meningiomas into 4 types. In our opinion, for Nauta type I and type II cystic IVMs, total resection should be performed as far as possible when the cyst walls are small and located in an unimportant functional area of the cortex, especially characterized by contrast enhancement. However, for Nauta type III and type IV cystic IVMs, if the cystic fluid is yellow or green and located around a nonfunctional region, the walls of the cyst should be removed as far as possible; nevertheless, partial residue is permissible if the cystic fluid is clear with cerebrospinal fluid and adjacent to functional structures, but regular follow-up is requisite.

## 4. Conclusions

We described 2 extremely rare cases of giant IVMs with cystic lesions, which may indicate the histopathologic malignancy of intraventricular meningioma. It is imperative to understand the continual updating of radiological and pathomorphological features of those patients for diagnostic and treatment purposes. Our report of the present paper may help to deepen the understanding of this rare clinical entity.

## Author contributions

**Conceptualization:** Hai Yu, Min Yang.

Data curation: Junhua He.

Methodology: Hai Yu, Junhua He, Min Yang.

Resources: Hai Yu.

Software: Junhua He.

Writing – original draft: Hai Yu, Junhua He.

Writing – review & editing: Min Yang.
